# Learning from digital health investments during COVID-19 vaccine program implementation: a research collaboration and theory of change

**DOI:** 10.1093/oodh/oqae005

**Published:** 2024-05-06

**Authors:** Nena do Nascimento, Amarynth Sichel, Adele Waugaman, Joy Kamunyori, Robert Rosenbaum, Jessica Shearer, Emily Carnahan, Caitlin Madevu-Matson, Eric Ramirez, Kevin Sakaguchi, Lauren Gilliss

**Affiliations:** Formerly Palladium, 1331 Pennsylvania Ave., NW, Suite 600, Washington, DC 20004, USA; Bureau for Global Health, United States Agency for International Development, GHTASC-PHI, 500 D Street SW, Washington, DC 20024, USA; Bureau for Global Health, United States Agency for International Development, GHTASC-PHI, 500 D Street SW, Washington, DC 20024, USA; Bureau for Global Health, United States Agency for International Development, GHTASC-Credence, 500 D Street SW, Washington, DC, 20024, USA; Bureau for Global Health, United States Agency for International Development, GHTASC-Credence, 500 D Street SW, Washington, DC, 20024, USA; PATH 455 Massachusetts Ave, NW, Washington, DC 20001, USA; PATH 2201 Westlake Ave., Suite 200, Seattle, WA 98121, USA; John Snow Inc. 1223, 44 Farnsworth St., #7, Boston, MA 02210, USA; Palladium, 1331 Pennsylvania Ave., NW, Suite 600, Washington, DC 20004, USA; Palladium, 1331 Pennsylvania Ave., NW, Suite 600, Washington, DC 20004, USA; John Snow Inc., 1223, 2733 Crystal Dr. 4th floor, Arlington, VA 22202, USA

**Keywords:** digital health, information systems, health system strengthening, COVID-19, pandemic, enabling environment

## Abstract

Responses to recent epidemics provide critical lessons on how the use of digital technologies and data systems can support timely and evidence-driven responses to public health emergencies. The arrival of COVID-19 and, subsequently, the COVID-19 vaccine, compelled many countries to attempt to create digitized, individual-level records on a large scale and quickly. In 2022, the United States Agency for International Development (USAID) brought together four global USAID-funded projects to explore whether and how USAID’s COVID-19 vaccine data and digital health investments supporting the emergency response strengthened the digital health enabling environment and, by extension, contributed to broader health system strengthening. Each project designed and implemented individual learning activities aligned to their specific USAID-funded COVID-19 vaccine response activities. The group collaboratively developed a theory of change to explore the potential relationship between COVID-19 vaccine-related digital and data investments and their immediate COVID-19 response, as well as two intermediate- and longer-term impact pathways: one focused on COVID-19-specific outcomes and impact, and a second focused on strengthening the digital health enabling environment and broader health system. The focus of this supplement is primarily to explore the theory of change associated with this latter pathway. Recognizing that health emergencies triggered by shocks due to disease outbreaks, climate change and conflict are likely to continue to characterize the environment in which health programs are delivered, this research seeks to contribute to a better understanding of how digital technologies and data systems can be most effectively leveraged to meet immediate needs while strengthening country resilience over the long term.

Abrégé

Les réponses aux récentes épidémies ont permis de tirer d’importants enseignements sur la façon dont le recours aux technologies numériques et aux systèmes de données peut appuyer le déclenchement rapide d’interventions fondées sur des éléments probants contre les urgences de santé publique. L’arrivée de la COVID-19 et, par la suite, celui du vaccin contre la COVID-19 ont contraint de nombreux pays à tenter de créer des dossiers numérisés au niveau individuel à grande échelle et rapidement. En 2022, l’United States Agency for International Development (USAID) a rassemblé quatre projets mondiaux qu’elle finançait dans le but d’explorer si et comment les données de l’USAID relatives au vaccin contre la COVID-19 ainsi que les investissements en santé numérique appuyant la réponse d’urgence avaient renforcé l’environnement propice à la santé numérique et, par extension, s’ils avaient contribué au renforcement du système de santé dans son ensemble. Chaque projet a conçu et mis en œuvre des activités d’apprentissage individuelles alignées sur leurs activités spécifiques de riposte au vaccin contre la COVID-19 financées par l’USAID. Le groupe a collaboré pour élaborer une théorie du changement afin d’étudier la relation potentielle entre les investissements dans le numérique et dans les données liées au vaccin contre la COVID-19 et leur réponse immédiate à la COVID-19 ainsi que deux itinéraires d’impact à moyen et à long terme: l’un était axé sur l’impact et les résultats spécifiques à la COVID-19 et le second sur le renforcement de l’environnement propice à la santé numérique et du système de santé dans son ensemble. Ce supplément a pour objectif principal d’examiner la théorie du changement associée à ce dernier itinéraire. Reconnaissant qu’il est probable que les conflits, les changements climatiques et les urgences sanitaires causées par des chocs dus à des épidémies continueront de caractériser l’environnement dans lequel sont offerts les programmes de santé, cette recherche vise à mieux faire comprendre la façon dont les technologies numériques et les systèmes de données peuvent être exploités le plus efficacement possible pour répondre aux besoins immédiats tout en renforçant la capacité de résilience des pays sur le long terme.

Resumen

Las respuestas a las epidemias recientes proporcionan lecciones críticas sobre cómo se puede apoyar respuestas oportunas y de base empírica a las emergencias de salud pública mediante el uso de tecnologías digitales y sistemas de datos. La llegada de la COVID-19 y, posteriormente, la vacuna contra esa enfermedad, obligó a muchos países a tratar de crear registros individuales digitalizados, a gran escala y rápidamente. En 2022, United States Agency for International Development (USAID) reunió cuatro proyectos globales que había financiado para estudiar si sus datos sobre la vacuna contra la COVID-19 y las inversiones en salud digital en apoyo de la respuesta de emergencia habían fortalecido el entorno propicio para la salud digital y, por extensión, si habían contribuido a fortalecer el sistema de salud en general, y de qué forma lo habían hecho. En cada proyecto se habían diseñado e implementado actividades de aprendizaje individuales, acordes con las actividades específicas de respuesta vacunal financiadas por USAID. El grupo desarrolló en forma colaborativa una teoría del cambio para analizar la posible relación entre las inversiones digitales y de datos y la vacuna contra la COVID-19 y su respuesta inmediata a la pandemia, así como dos vías de impacto a mediano y largo plazo: una primera centrada en los resultados y efectos específicos en la pandemia, y una segunda centrada en fortalecer el entorno propicio para la salud digital y un sistema de salud más amplio. El enfoque de este suplemento consiste principalmente en examinar la teoría del cambio vinculada con esta última vía. Reconociendo que es probable que las emergencias sanitarias provocadas por los brotes de enfermedades, el cambio climático y los conflictos continúen caracterizando el entorno en el que se ejecutan los programas de salud, esta investigación busca ayudar a que se comprenda mejor la forma en que las tecnologías digitales y los sistemas de datos pueden aprovecharse de manera más efectiva para satisfacer las necesidades inmediatas, fortaleciendo la resiliencia de los países a largo plazo.

## BACKGROUND

Responses to recent epidemics provide critical lessons on how the use of digital technologies and data systems can support timely and evidence-driven responses to public health emergencies. During the 2014–17 West Africa Ebola epidemic, data use and information were critical components of the epidemic response [1]. Specifically, digital technologies provided predictions and estimations for transmission, clinical workforce training and development and community surveillance and mobilization [2–4]. However, the use of digital technologies and data systems was not without significant challenges. Delays in transferring data from paper to digital records at times thwarted timely decision-making [1]. There were also notable coordination challenges around the use of digital systems, a lack of alignment around data standards and a lack of interoperability across systems, which together hindered the ability of governments and programs to collect and use data to bolster the outbreak response [1]. Moreover, a significant proportion of investments during the West Africa Ebola outbreak were made in custom-built systems that failed to scale or be sustained beyond the emergency phase of the response [1].

The global AIDS epidemic response has likewise provided valuable lessons and strategies for deploying digital health solutions for public health impact. The four decades of experience have reinforced the importance of developing electronic medical record-keeping systems and aggregate information systems for timely and accurate data for clinical and community service delivery and program management [5]. As compared to other health areas, HIV programs have more actively pursued and scaled individual-level client records, supported by significant funding outlays. That said, critics have noted the often-siloed nature of HIV information systems, the relative discrepancy in investment in information systems across other non-HIV-related health areas in some countries, and the need to better integrate health data systems to avoid the proliferation of parallel reporting systems and structures [6, 7].

As the world turned its attention in 2021 to tackling COVID-19 through vaccination programs of historic proportions, digital tools once again played an important role in supporting the emergency response. In many countries, this represented a change in immunization processes. For example, although client-level routine immunization data had been digitized to some extent, these data had largely been recorded and stored on paper, and mostly for small subsets of the population (e.g. children of certain ages). To drive the population-scale vaccination effort, countries and their health system partners leveraged digital tools and data systems, including for planning and management, cold chain and supply logistics, service delivery, monitoring, risk communication and engagement and training and human resources for health.

**Figure 1:**
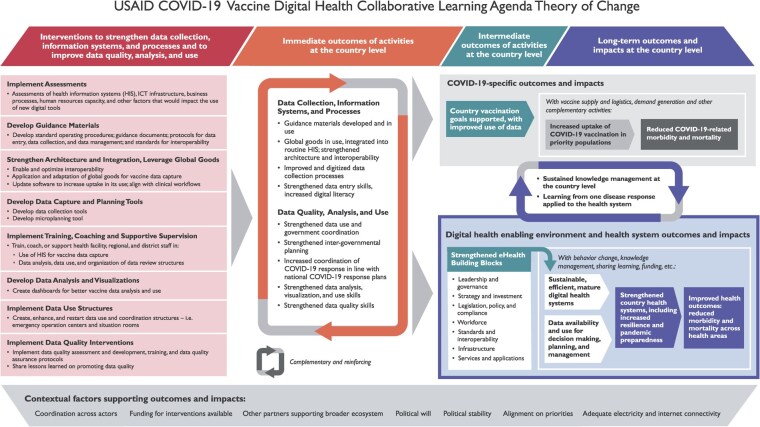
USAID COVID-19 vaccine digital health collaborative learning agenda theory of change

The United States Agency for International Development (USAID) was one of the main funders of the global COVID-19 vaccination effort and made significant digital health and data system investments to support this work. USAID has policies, informed by lessons learned from the Ebola, HIV, malaria, tuberculosis and other global health responses, that guide its approach to digital health investments in partner countries. These include USAID’s *Vision for Action in Digital Health 2020–2024*, *Digital Strategy 2020–2024* and *Vision for Health System Strengthening 2030* [8–10].

To better understand how these policies may have influenced the work of USAID-funded partner organizations implementing emergency COVID-19 vaccination digital and data system investments, and to capture lessons to further refine its approach, in 2022 USAID brought together four global USAID-funded projects: Data for Implementation (Data.FI), Country Health Information Systems and Data Use (CHISU), MOMENTUM-Routine Immunization Transformation and Equity (M-RITE) and Digital Square. USAID asked these projects to conduct a series of learning activities across a range of countries (Burkina Faso, the Democratic Republic of the Congo, Guatemala, Honduras, Indonesia, Kenya, Mali, Niger, Senegal, Suriname, Tanzania and Vietnam) to explore whether and how USAID’s COVID-19 data and digital health investments supporting the emergency response contributed to strengthening the digital health enabling environment and, by extension, broader health system strengthening. Data.FI served as the convening partner for this learning group.

The group of projects and learning activities, referred to as the ‘COVID-19 Vaccine Digital Collaborative Learning Agenda’ (or ‘Learning Agenda’), jointly addressed a set of key learning questions to determine the extent to which investments during the COVID-19 response were supportive of (or not supportive of) longer-term changes in the digital health enabling environment. The group collaboratively developed a theory of change (Fig. 1), the subject of this paper, to chart the potential relationship between COVID-19 vaccine-related digital and data investments and improved outcomes and impacts for the digital health enabling environment and broader health system. The aim was to identify lessons that could be immediately used as countries transitioned their emergency COVID-19 vaccine-related digital tools and systems into routine health systems, to help prepare for future pandemics and to develop recommendations for how the global health community can more effectively and sustainably deploy digital health and data solutions in future health emergencies.

**Table 1:** Summary of the learning agenda supplement articles and associated learning agenda questions

**Authors**	**Article name**	**Countries**	**Global goods utilized?**	**Learning question(s) answered**
Carnahan *et al*.	Root causes of COVID-19 data backlogs: a mixed-methods analysis in four African countries	Democratic Republic of the Congo (DRC), Kenya, Senegal and Tanzania	Yes – DHIS2 was used in all four countries. Also studied was Chanjo Kenya, a custom-built electronic immunization registry system built by the Kenya Ministry of Health and Ministry of Information, Communication, and Technology.	How, and to what extent, did USAID’s COVID-19 health system, data and digital health investments contribute to a strengthened digital health enabling environment as defined by the seven WHO/ITU eHealth building blocks? How, and to what extent, did USAID’s COVID-19 health system, data and digital health investments leverage existing or develop new global goods, resulting in standard operational, functional and system requirements that advance country replication and/or increase global digital and data system investment repositories?
Boyle *et al.*	Does investment in COVID-19 information systems strengthen national digital health architecture? Lessons learned from Burkina Faso, Indonesia, Mali and Suriname	Burkina Faso, Indonesia, Mali and Suriname	Partially – DHIS2 and DHIS2 Tracker were used in Burkina Faso, Mali and Suriname for COVID-19 vaccination tracking. Indonesia developed and deployed its own COVID-19 vaccine digital tools.	To what extent and how have USAID’s COVID-19 health system, data and digital health investments advanced the country’s digital health architecture by lowering the financial and management burden of competing digital systems and by improving interoperability between national digital health systems?
Hidalgo *et al.*	Implementation of a data use strategy in situation rooms in two metropolitan areas of Honduras in the context of COVID-19	Honduras	No – while the government tested use of DHIS2 Tracker, ultimately global goods were not part of the COVID-19 vaccine digital tracking response. The SIVAC aggregated immunization system already in place was utilized.	How and to what extent, did USAID’s COVID-19 health system, data and digital health investments contribute to a strengthened digital health enabling environment as defined by the seven WHO/ITU eHealth building blocks?
Mpanya *et al.*	Interventions and adaptations to strengthen data quality and use for COVID-19 vaccination: a mixed methods evaluation	DRC, Niger and Vietnam	Partially – DHIS2 Tracker was used in DRC; Vietnam deployed MS Word and Excel-based tracking systems. In Niger, COVID-19 vaccine tracking tools were not studied; rather, a proprietary remote temperature monitoring solution was evaluated for the vaccine cold chain.	How and to what extent, did USAID’s COVID-19 health system, data and digital health investments contribute to a strengthened digital health enabling environment as defined by the seven WHO/ITU eHealth building blocks?
Gilliss *et al.*	How do countries select and use digital global goods in emergency settings? Lessons learned from the DHIS2 COVID-19 data management experiences in Burkina Faso, Mali and Suriname	Burkina Faso, Mali and Suriname	Yes – DHIS2, DHIS2 Tracker and CommCare mobile solutions were used in the three countries.	How, and to what extent, did USAID’s COVID-19 health system, data and digital health investments leverage existing or develop new global goods, resulting in standard operational, functional and system requirements that advance country replication and/or increase global digital and data system investment repositories?

The group’s learning questions were: How, and to what extent, did USAID’s COVID-19 health system, data and digital health investments:

- Contribute to a **strengthened digital health enabling environment** as defined by the seven World Health Organization/International Telecommunication Union (WHO/ITU) eHealth building blocks?
- Support a **government-led, coordinated approach** to addressing needs identified by the country’s digital health strategy and costed implementation plans (or other organizing policy, coordination structure and/or budgeting framework)?
- Advance the **country’s digital health architecture** by lowering the financial and management burden of competing digital systems and by improving interoperability between national digital health systems?
- Leverage existing or develop new **global goods**, resulting in standard operational, functional and system requirements that advance country replication and/or increase global digital and data system investment repositories?

## METHODS

The initial set of learning questions was developed by USAID team members to assess alignment of the projects’ COVID-19 response with USAID’s *Vision for Action in Digital Health 2020–2024* [8]. The learning questions were refined by USAID following discussion with, and agreement by, the Learning Agenda group on the definitions of key terms (described subsequently). Each project designed and delved into tailored learning questions aligned to their USAID-funded COVID-19 vaccine response activities.

A theory of change was developed iteratively based initially on each project’s activity-specific, country-level theories of change. Once the individual theories of change were developed, they were reviewed, aligned and consolidated to create one synthesized framework that reflected the group’s joint efforts. The consolidation was originally undertaken by Data.FI, and then refined and aligned during and following an all-day workshop in November 2022 with all Learning Agenda group participants. Components of the theory of change were intended to be used to test the interventions studied and ground the findings of all papers in this supplement with the understanding that (1) any individual activity would not touch on all aspects of the theory of change framework and (2) it would not be feasible within the time frame to measure the longer term outcomes and impacts of the interventions studied.

Members of the Learning Agenda group met monthly for 1 year (August 2022–August 2023) to coordinate this joint learning activity, to discuss progress on the learning activities and to identify common themes and potential areas of current or future collaboration. The result of this collaboration was the development of a journal supplement which consists of: (1) this article, which provides the theoretical framing for this collaboration and journal supplement; (2) five articles that summarize individual learning activities, findings and how they align with the theory of change presented in this article and one article that describes a framework for leveraging investments in COVID-19 immunization systems; and (3) a commentary, which provides broader discussion on the implications of these findings and the future direction of digital health interventions during an emergency response.

The studies in this supplement include work that spanned across 11 countries on three continents. While they comprise a relatively small part of USAID’s overall contribution to the US Government’s Initiative for Global Vaccine Access, they were selected as programs focused specifically on data and digital health systems as part of a coordinated effort launched in early 2022. The five articles that represent the learning activities, countries where the learning activities took place, to what extent global goods were studied or part of implementation and associated learning questions answered are presented in Table 1.

### Definitions of key terms

Digital health—the use of information and communication technology (ICT) to support health and well-being—is critical to integrated, resilient and responsive health systems [11]. For digital health to sustainably contribute to health systems, its implementation must be supported by an **enabling environment.**

WHO’s *National eHealth Strategy Toolkit* describes the environmental components or ‘building blocks’ required for digital health interventions to effectively and sustainably contribute to the health system. These building blocks consist of leadership and governance; strategy and investment; legislation, policy and compliance; workforce; infrastructure; standards and interoperability; and services and applications. When strategically implemented and developed, the building blocks allow the development, implementation, maintenance and enhancement of digital health interventions and solutions for health service delivery [12].

As part of the collaboration, the Learning Agenda group jointly defined several other key terms from the learning questions, using the existing literature, while recognizing that these definitions describe an ideal state and may not be achieved in all settings.

**Digital health enabling environment:** An enabling environment is a construct described by WHO in 2019 as ‘… the attitudes, actions, policies and practices that support the effective and efficient functioning of organizations and programmes’ [13]. The digital health enabling environment is made up of the seven eHealth building blocks described in WHO’s National eHealth Strategy Toolkit.

**Government-led, coordinated approach:** Country governments direct and coordinate digital COVID-19 activities at the national level, in close coordination with donors and implementing partners, to ensure that there is alignment with broader strategic and health goals, existing health and digital health strategies and costed implementation plans—and that there is political support and complementary, rather than duplicative, digital health activities to strengthen the pandemic response [8].

**Country digital health architecture**: A country’s digital health architecture provides a blueprint or roadmap of data, systems, technologies, standards and guidelines to identify country-specific technology and governance requirements that can enable interoperability among national digital health systems and their ability to integrate digital technologies using shared services. National digital health architectures can lower the financial and management burden of competing digital systems through the use of reusable systems or assets; strengthen national health institutions and the provision of health care overall; and promote the effectiveness, reach and cost-efficiency of digital investments [8, 11, 14].

**Global goods**: Global goods are tools that are adaptable to different countries and contexts to help address key health system challenges. There are three types of global goods in the health sector: (1) content (resources, toolkits or data standards), (2) software and (3) services. They are open-source, adaptable and reusable to meet the diverging needs of various geographic, thematic contexts and digital maturity levels. Mature global goods are free and open-source, supported by a strong community, used across multiple countries, have demonstrated effectiveness, are designed to be interoperable and are emergent standard applications [8, 15, 16].

### Description of the learning agenda theory of change

Through individual and collaborative work, the Learning Agenda group developed and refined a theory of change that mapped the potential relationship between USAID’s COVID-19 vaccination data and digital investments and any potential outputs, outcomes and impacts related to strengthening the digital health enabling environment and health system in the countries of focus.

The emergency-funded digital and data interventions supported by USAID-funded partners were designed and implemented through collaboration with government partners in support of country priorities. They are described on the left of the theory of change diagram (see Fig. 1) and are grouped by intervention area. We hypothesized these interventions would contribute to the immediate outcomes described in the middle of the diagram. These immediate outcomes—grouped as ‘Data Collection, Information Systems and Processes’ and ‘Data Quality, Analysis and Use’—pertain specifically to COVID-19 and are short-term. Learning Agenda members expected to be able to measure and quantify these outcomes within the course of 1 year. The Learning Agenda group members recognized that there is overlap between the two immediate outcome categories, which are complementary and mutually reinforcing.

The Learning Agenda group further hypothesized that the immediate outcomes of the interventions would contribute to two impact pathways—one focused on COVID-19-specific outcomes and impact, and a second focused on outcomes and impacts related to strengthening the digital health enabling environment and health systems more broadly. As with the immediate outcomes, the two longer-term impact pathways—although divergent in the diagram—are understood by the group to have the capacity to be complementary and mutually reinforcing, particularly when designed with intentionality. While the Learning Agenda group was interested in understanding the relationship among the interventions, immediate outcomes and the COVID-19-specific outcomes and impacts, the group focused primarily on understanding the second pathway—to better understand to what extent and how COVID-19 vaccine investments are supportive of a strengthened digital health enabling environment and, by extension, health system. A description of each component of the theory of change follows.

**Interventions**: The interventions undertaken by the four projects represented in the Learning Agenda group were intended to strengthen COVID-19 vaccination data (e.g. doses administered, vaccine supply, temperature at which vaccines are stored, target population(s) and their needs and other areas pertaining to COVID-19 vaccination). These interventions were designed to deliver immediate outcomes of improving country-level (i) data collection, information systems and processes, and (ii) data quality, analysis and use. These interventions, listed in the left-hand column in the theory of change (Fig. 1), should not be taken as stand-alone, one-off interventions—most projects implemented their work across multiple categories. For example, an assessment was often a prerequisite for training, coaching and supportive supervision activities, or for the adaptation of global goods for data collection.

We grouped the interventions in the following categories:

- **Implementation of assessments** to better understand and diagnose interventions and activities in the health information system (HIS), ICT infrastructure, business processes, human resources and other factors that would impact the use of digital tools.
- **Development of guidance materials**, including standards for interoperability, standard operating procedures for data collection, data entry and data management, etc.
- **Strengthening digital health architecture** and digital systems integration through: enabling and optimizing interoperability, supporting the application and adaptation of global goods and updating software to increase use and better align to clinical workflows.
- **Development and/or refinement of data capture** and planning tools for COVID-19 vaccine implementation.
- **Training, coaching and supportive supervision** for health facility, district and/or regional ministry of health staff in the use of an HIS for COVID-19 vaccine data capture, and in data analysis, data use and organization of data review structures.
- **Development of data analysis and visualizations** for COVID-19 vaccine data to support data use by ministry of health staff.
- **Creation and support of data review and coordination structures** to facilitate the use of data for the COVID-19 vaccine response.
- **Design and execution of interventions** to strengthen the quality of COVID-19 vaccine data.

**Immediate Outcomes**: As previously mentioned, the immediate outcomes of the interventions are described in the middle of the theory of change (Fig. 1) and are divided into two main areas, which are complementary and reinforcing: first, strengthened data collection and information systems and processes; and second, improved data quality, analysis and use. The immediate outcomes of strengthened data collection, improved information systems and better business processes can lead to better quality data, more available data and greater use of data (and vice versa). This value chain can benefit both immediate priorities, in this case supporting the effective, targeted delivery of COVID-19 vaccines, in addition to contributing to longer-term health program and health system impacts.

The Learning Agenda group’s theory of change posits that investments in: improved data collection processes; strategically identified digital systems; and strengthened processes for data collection, data management and reporting will make information for decision-making more available to a range of stakeholders. When fit-for-purpose data are more available, they can be better used, and more use of data can drive improvements in data quality, and, by extension, contribute to strengthened health program planning, management and delivery [17–20]. Alternatively, starting with the ‘Data Quality, Availability and Use’ grouping: when there are improved coordination and data review structures in place to use data, there is more demand for higher-quality data for decision-making, which then drives recommendations to strengthen data collection processes and information systems as well as strengthen business processes that support these systems.

Immediate outcomes related to data collection and information systems/processes include the availability and use of guidance materials; use of global goods that are integrated into the routine HIS; strengthened HIS architecture and interoperability across systems; improved and digitized data collection processes; strengthened data entry skills; and increased digital literacy for data collection, data entry and HIS use. Immediate outcomes under data quality, data analysis and data use include strengthened use of data for decision-making by ministry of health staff; strengthened inter-governmental planning; improved government coordination to support the pandemic response in line with COVID-19 response plans; strengthened skills of government staff in data analysis, visualization and use; and strengthened data quality practices and procedures.

**Intermediate Outcomes and Long-Term Outcomes and Impact:** There are two sets of intermediate outcomes in the theory of change (Fig. 1). The first pathway pertains to supporting country COVID-19 vaccination goals through improved use of data. While this pathway was not the primary pathway of interest for this research, it is the primary route to impact that most activities in this supplement were designed to follow. The improved use of data is the result of the immediate outcomes described above. The theory of change posits that by supporting country vaccination goals (e.g. vaccination targets across the population, including among specific priority populations) with the improved use of data, there could be increased uptake of COVID-19 vaccination in priority populations, which in turn could lead to reduced COVID-19-related morbidity and mortality [21]. This sequence of events assumes that the vaccines are effective (e.g. they have not experienced temperature excursions that alter their efficacy), available and accessible, which requires complementary activities, including demand generation and supply chain management.

The theory of change further hypothesizes that, when supported by digital health interventions that have been intentionally designed to support cross-cutting health system strengthening, the immediate outcomes in turn can be supportive of the broader digital health enabling environment, which eventually can result in broader health system strengthening in a given country context. (The complementary and mutually reinforcing arrow between the two outcome and impact pathways illustrates this.) Strengthening the building blocks of a country’s digital health enabling environment can enable digital investments to support routine immunization and other health areas, including the use of data for routine health service delivery and to prepare for and respond to future pandemics [22, 23].

**Contextual Factors Supporting Outcomes and Impact**: The theory of change includes key cross-cutting contextual factors, listed at the bottom of Fig. 1, which influence implementation of the interventions and the extent to which they can lead to the expected outcomes and impact. These are:

- **Coordination among key development actors in a country and in the broader donor community** to ensure that digital health investments are aligned and complementary such that all partners are working together to achieve similar goals.
- **Availability of funding for current and future investments** in digital health and public health to ensure that the implementation of activities is possible and can be sustained in the longer term.
- **Existence of additional partner support of the broader digital and public health ecosystem,** recognizing that the sum of the efforts described here are not exhaustive and must be supported by other partners to strengthen elements of the digital and public health ecosystem simultaneously and over time.
- **Government political will and alignment from implementing partners and donors to country public health and digital health priorities** to ensure that the objectives for strengthening the enabling environment and health system strengthening are supported and sustained.
- **Country political stability** so that activities and investments made are not abruptly halted or unable to proceed due to changes in government leadership, conflict or an otherwise volatile environment.
- **Adequate infrastructure**, including electricity and connectivity, for digital health interventions to be viable and at scale.

These contextual factors describe elements of an enabling environment that influence the success of digital health interventions.

## DISCUSSION

This theory of change is informed by the understanding that experiences from one health response can be and are leveraged to address another. Studies have shown that the AIDS crisis of the 1980s and 90s led to lessons learned and a more rapid response during the more recent Ebola and Zika outbreaks in sub-Saharan Africa and Latin America and the Caribbean [24, 25]. There is early evidence that African countries were more responsive and resilient to the COVID-19 pandemic than some countries in other regions because of the decades of investment in public health programming across the continent, especially through the US President’s Emergency Plan for AIDS Relief investments over the past 20 years [26–28]. The development of the Learning Agenda group’s theory of change highlights the extent to which the WHO/ITU eHealth building block framework continues to be a relevant, durable and readily applicable tool for conceptualizing digital health implementation at a national level. That said, the use of this framework for the creation of this theory of change also highlights specific aspects of the framework that are not well articulated.

First, there is a lack of clarity about how to address and integrate data use and data use interventions in the framework. Although actors that use data are included in the framework under ‘workforce’ and ‘leadership’, data use is not called out as a separate building block, which made it challenging to depict data use in the theory of change. Second, in its discussions, the Learning Agenda group articulated that widespread behavior change and change management processes were needed in a health system to ensure that the interventions supporting COVID-19 vaccine digital response were successful. This was especially significant given that several activities implemented and studied by this group focused on digitizing paper COVID-19 vaccine records. The behavior change/system-wide change management process could be understood to be a cross-cutting driver in the existing enabling environment (under workforce, leadership, etc.), or it could be thought of separately and in a way not currently explored by this model.

These first two observations align with research from Werner *et al*., which explores the digitalization process in five countries in sub-Saharan Africa [29]. They suggest expanding the existing WHO/ITU framework to include (1) cultivating a culture of data use (which is comprised of data collection, management, analysis and dissemination) and feedback on data and data use (and data quality and accessibility to relevant users); and (2) change management to manage the ‘system-wide behavior change required to move from manual or paper-based to digital systems’.

The Learning Agenda group also considered the roles of community culture, organizational culture and organizational structures during intervention development and implementation [30]. Although there is currently limited literature on these topics, there is growing documented evidence of the role of gender and culture in influencing the acceptability and use of digital solutions, and the importance of adapting digital health tools to the cultural context [31, 32]. This was an important factor during the Ebola outbreak in West Africa; similarly, the influence of gender and culture has been documented as it relates to acceptability of the COVID-19 vaccine [1, 33].

## CONCLUSION

Building a better understanding of the mechanisms by which digital tools are successful in supporting health systems during a shock is critical given the uncertain future we are facing across the world and the high levels of interest in leveraging advanced technological solutions, such as artificial intelligence and the digital transformation movement, for health. With the emergence of COVID-19, for the first time, countries attempted to create digitized individual-level records on a large scale (for their entire populations) and quickly.

The authors’ development and exploration of this theory of change through the studies presented in this supplement can support decision-makers, health implementers, donors, government stakeholders and technologists to better design and implement digital health interventions during a health emergency. The studies also provide lessons for strengthening routine HIS and broader health system strengthening, with a greater focus on how to support a stronger digital health enabling environment and strengthened systems, even in the midst of a crisis. We recommend this collaborative learning model with a shared theory of change be considered by others as an expanded approach for large, complex global health initiatives and other complex global challenges to facilitate better alignment and coordination, improve resource efficiency and reduce burden on individual country governments.

## Data Availability

No new data were generated or analyzed in support of this research.
